# Silver Nanowire Electrodes: Conductivity Improvement Without Post-treatment and Application in Capacitive Pressure Sensors

**DOI:** 10.1007/s40820-014-0018-0

**Published:** 2014-11-14

**Authors:** Jun Wang, Jinting Jiu, Teppei Araki, Masaya Nogi, Tohru Sugahara, Shijo Nagao, Hirotaka Koga, Peng He, Katsuaki Suganuma

**Affiliations:** 1 State Key Laboratory of Advanced Welding and Joining, Harbin Institute of Technology, Harbin, 150001 People’s Republic of China; 2 The Institute of Scientific and Industrial Research, Osaka University, Osaka, Ibaraki 567-0047 Japan

**Keywords:** Silver nanowire, Pre-treatment, Transparent electrode, Pressure sensor

## Abstract

Transparent electrode based on silver nanowires (AgNWs) emerges as an outstanding alternative of indium tin oxide film especially for flexible electronics. However, the conductivity of AgNWs transparent electrode is still dramatically limited by the contact resistance between nanowires at high transmittance. Polyvinylpyrrolidone (PVP) layer adsorbed on the nanowire surface acts as an electrically insulating barrier at wire–wire junctions, and some devastating post-treatment methods are proposed to reduce or eliminate PVP layer, which usually limit the application of the substrates susceptible to heat or pressure and burden the fabrication with high-cost, time-consuming, or inefficient processes. In this work, a simple and rapid pre-treatment washing method was proposed to reduce the thickness of PVP layer from 13.19 to 0.96 nm and improve the contact between wires. AgNW electrodes with sheet resistances of 15.6 and 204 Ω sq^−1^ have been achieved at transmittances of 90 and 97.5 %, respectively. This method avoided any post-treatments and popularized the application of high-performance AgNW transparent electrode on more substrates. The improved AgNWs were successfully employed in a capacitive pressure sensor with high transparency, sensitivity, and reproducibility.

**Electronic supplementary material:**

The online version of this article (doi:10.1007/s40820-014-0018-0) contains supplementary material, which is available to authorized users.

## Introduction

Transparent electrodes are regarded as essential components in optoelectronic applications such as solar cells, touch screens, organic light-emitting diodes, and sensor devices [1–4], and indium tin oxide (ITO) thin films are the most widely used material for such applications. However, there are several drawbacks to use ITO thin film, such as the inherent brittleness, the expensive deposition process, and also the emerging indium scarcity. Several alternatives have been investigated, including carbon nanotubes (CNTs), graphene, conductive polymer, and metal nanowires [5–7]. Among these, transparent electrode based on silver nanowire (AgNW) networks is being studied intensively and attracting commercial interest owing to their great potential for flexible, cost-efficient, and large-scale fabrication [8–11].

Although bulk silver exhibits very low electrical resistivity, the conductivity of AgNW networks is limited, especially at high transmittance, by the contact resistance between wires due to the residual of polyvinylpyrrolidone (PVP) layer, which is usually employed as the capping agent to control nanostructure size and disperse nanowires during AgNW synthesis [12, 13]. Several methods have been developed to enhance the contact between nanowires, such as high-temperature (above 200 °C) or long-duration thermal annealing [14, 15], rinsing and pressure treatment [16], and photonic sintering [17]. Other techniques such as nanosoldering, microwire enhancement, and galvanic displacement aim to remove the PVP layer or enlarge the contact area at wire–wire junctions [3, 18, 19]. However, all of these are the post-treatment methods after AgNWs are deposited on the substrate or the surface of device. Therefore, not only do these methods complicate the fabrication process, they also inevitably influence the performance of the heat-sensitive, pressure-sensitive, or chemical-sensitive substrates.

Recently, a new strategy was proposed using long nanowires to reduce the number of wire–wire junctions in conductive paths, thereby leading to low sheet resistance [20, 21]. However, the PVP-induced resistance problem still has not been solved. The residual PVP layer adsorbing on the surface of AgNW still acts as an electrically insulating barrier at the wire–wire junctions and undermines the conductivity of the electrode. As the saying goes, “sharpen the knife before cutting the wood,” i.e., high-performance AgNW ink is prerequisite before the fabrication of AgNW transparent electrode. Hence, decreasing the thickness of PVP layer beforehand will be a suitable, simple, and effective method for conductivity improvement. Unfortunately, many efforts are still focused on various post-treatments of AgNW electrodes as mentioned above. So far, few reports have paid attention to the important process to improve AgNW ink before electrode fabrication. AgNW paste washed by water has been reported to joint copper at room temperature without pressure [22], but this close-packed AgNW layer with thickness over 20 μm was quite different from transparent electrode. The actual effect of filtration washing and sonication dispersing process could improve the conductivity of AgNW films [15]; however, it is too time-consuming to produce nanoscale ink by filtration in practical applications. Moreover, the washing effect on long AgNW has not been discussed. In this paper, a simple and rapid washing method is proposed to tailor the thickness of PVP layer on the surface of very long AgNWs, and the nanowire ink for high-performance transparent electrode regardless of substrate properties was achieved accordingly. Solvents and washing parameters were carefully selected and adjusted to meet the dispersion and preservation requirements of the nanowires. Finally, as-washed AgNWs were used in capacitive pressure sensor, which showed high transparency, sensitivity, and reproducibility.

## Experimental

### Preparation of AgNWs

AgNWs for the preparation of transparent electrodes were synthesized using a one-step polyol method starting with two solutions. For the first, 1.0 g silver nitrate was dissolved in 40 mL ethylene glycol at room temperature, while for the second, 0.8 g PVP (Mw = 360,000) was gradually dissolved in 50 mL ethylene glycol at 60 °C while stirring at 300 rpm. After complete dissolution, the two solutions were mixed and 13.6 g of FeCl_3_ solution (600 μmol L^−1^ in ethylene glycol) was added to the mixture at room temperature and stirred at 300 rpm for 3 min. The mixture was then heated at 110 °C without stirring for a 12-h redox reaction. Finally, the solution was mixed with acetone at a volume ratio of 1:4 to precipitate AgNWs for the subsequent steps.

### Washing Method

Thickness tailoring of PVP nanolayer was performed by first mixing AgNW precipitate with ethanol at a mass ratio of 1:15 and stirring at 150 rpm for 15 min at room temperature, followed by centrifugation of the suspension at 3,000 rpm for 3 min. The supernatant was carefully decanted, and the residual precipitate was dispersed in ethanol and prepared for further washing. This procedure constitutes one cycle of ethanol washing (E1) and was applied twice, thrice, or four times to obtain three more AgNW ink-labeled E2, E3, and E4. Then, E4-AgNWs were mixed with deionized (DI) water at either 25 or 90 °C, with stirring at 150 rpm for 15 min, to obtain W25-AgNWs and W90-AgNWs, respectively. Meanwhile, E4-AgNWs were also mixed with dimethylformide (DMF), at either 25 or 140 °C, with stirring at 150 rpm for 15 min to form D25-AgNWs and D140-AgNWs, respectively. The ink in DI water and DMF were then centrifuged at 3,000 rpm for 3 min to obtain AgNW precipitate. Since the wetting properties of these three solvents on PET were different, the AgNWs were finally dispersed in ethanol for coating. All these AgNW inks were fixed at 1.2 wt % concentration. All the reagents mentioned above were purchased from Wako Pure Chemical Industries, Ltd.

### Fabrication of AgNW Transparent Electrodes

PET films with a thickness of 100 μm were employed as substrates for AgNW electrodes. The PET substrates were cleaned in ethanol with ultrasonic treatment and then dried in air. The well-dispersed AgNW inks were drop-coated on the PET substrates for various transmittances at 550 nm wavelength. The dropped ink spread on the surface until uniformly coating the substrate, and the specimens were dried in air for 2–3 min until the solvent is evaporated. AgNW ink was also coated on glass beaker, PET bottle, tissue paper, and bacterial cellulose to verify its adaptability on various substrates. Coating method for glass beaker and PET bottle was dip-coating, and for tissue paper and bacterial cellulose was also drop-coating.

### Fabrication of Pressure Sensors

AgNW transparent electrodes on PET substrates were employed in capacitive pressure sensors. Pure PVP was dissolved in ethanol at 5 wt % concentration. The PVP solution was spin-coated on the as-prepared AgNW electrodes at room temperature. After drying in air for about 30 s, two pieces of sandwiched PET/AgNWs/PVP structures were assembled with the two PVP layers in contact. Two pieces of conductive tapes were pasted on AgNW electrodes for the capacitance measurement.

### Measurements and Characterization

The morphology of the PVP layer adsorbed on the AgNWs was investigated by optical microscope (VHX-600, Keyence), scanning electron microscope (SEM, SU8020, Hitachi High-Technologies), and transmission electron microscope (TEM, JEM-2100, JEOL). The sheet resistance of 30 mm × 20 mm AgNW electrodes was measured using a four-point probe meter (Loresta GP T610, Mitsubishi Chemical Analytech). The transmittance investigated here was the transmittance of parallel light and does not include the transmittance of diffused light. The parallel transmittance (*T*_p_) of the AgNW electrode for wavelengths in the range 300–900 nm was measured using a UV–visible/near-infrared spectrophotometer (V670, JASCO). The testing window size of the spectrophotometer is 12 mm × 6 mm, and three different places of one sample were tested and the average value was used as the transmittance for each sample. Thermogravimetry analysis (TGA) was carried out on a thermal analyzer (TG–DTA 2000SA, Netzsch Japan). The electrode haze was measured using a D65 illumination haze meter with a strong visible light source (HZ-V3, Suga Test Instruments). For the capacitive sensor, changes in capacitance were measured using a digital multimeter (34410A, Agilent Technologies).

## Results and Discussion

**Fig. 1:**
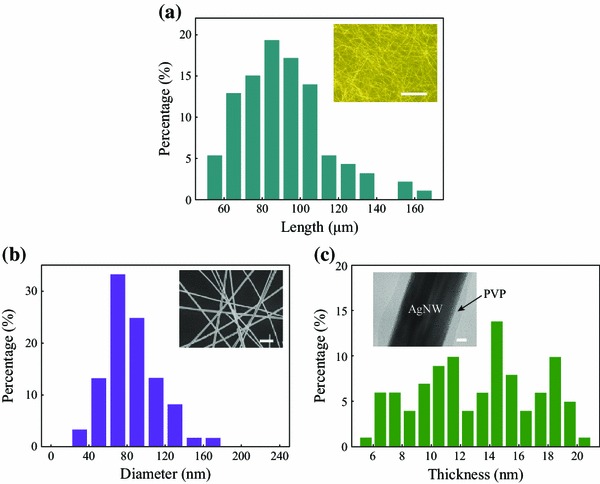
AgNWs were successfully synthesized by one-step polyol method using PVP as a capping agent. **a**,**b** The length and diameter distribution of the as-synthesized AgNWs.*Inset*: morphology of AgNWs, low resolution (*Scale bar* = 50 μm) and high resolution (*Scale bar* = 1 μm). **c** The thickness distribution of PVP nanolayer on the surface of AgNWs.*Inset*: the TEM image of individual AgNW with PVP nanolayer. *Scale bar* = 20 nm

Transparent electrodes are designed to achieve both outstanding electrical conductivity and optical transmittance; however, these two properties are seemingly negatively correlated, since the higher density of nanowires leads to the increase of conductivity and also the decrease of transparency. This dilemma could be resolved by employing long AgNWs. For the one-step wet chemistry method, the length and diameter of AgNWs varied drastically with the reaction temperature and the stirring speed [12]. In this study, the procedure for growing AgNWs was modified as at low temperature of 110 °C without stirring. The as-synthesized AgNWs, shown in Fig. 1, had an average length of 89.5 μm and an average diameter of 84.2 nm. During the synthesis and growth of AgNWs, the PVP capping agent was gradually adsorbed on the surface of the newly emerging silver crystals and grew with the crystals to be a nanolayer on the surface of the nanowire, formed from the merging of the crystals (Fig. 1c inset). The thickness of the nanolayer ranged from 5.96 to 20.13 nm with an average of 13.19 nm (Fig. 1c). The PVP layer prevents the agglomeration of AgNWs due to steric repulsion [23], while too thick layer also increases the contact resistance. Therefore, the thickness of PVP layer needs to be tailored appropriately.

**Fig. 2:**
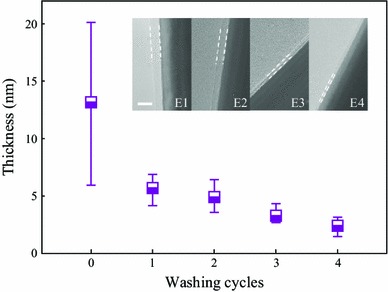
Average thickness distribution of PVP nanolayer.*Inset*: TEM images of AgNWs washed with ethanol for different cycles. *Scale bar* = 10 nm

Many post-processing methods, such as heating and pressing, have been proposed to thin down the PVP layer. In fact, it is known that PVP dissolves well in various solvents such as water, methanol, ethanol, acetic acid, chloroform, and so on [24]. Therefore, it is feasible to use these solvents to reduce the thickness of PVP layer. In this study, a simple stirring, washing, and centrifugation method was employed, and its effect on the thickness of the PVP layer was investigated. Figure 2 shows that the average thickness is dramatically reduced to 5.74 nm after a single washing cycle using ethanol and then decreased gradually down to 2.43 nm after four cycles. The TEM images shown in the inset of Fig. 2 highlight the thickness change of PVP layer on the surface of AgNWs during washing. The thickness visibly decreased compared with that of the original AgNWs and gradually thinned after each washing cycle. This trend is also verified by the thermogravimetric analysis (TGA) shown in Fig. S1. For the untreated AgNWs samples, the weight loss indicates intense evaporation of organic residues. The rapid weight loss occurring between ~300 and 450 °C was attributed to the evaporation and decomposition of residual byproducts and PVP [22]. For E4-AgNWs, the weight loss at high temperature is much less, demonstrating that after four cycles of washing, a mass of PVP and byproducts had been removed.

**Fig. 3:**
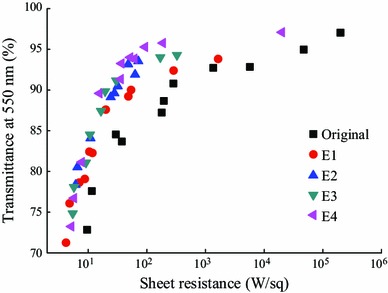
Sheet resistance of AgNW electrodes with transmittances at 550 nm wavelength. Electrodes with original AgNWs were applied to compare with the ones with AgNWs washed in ethanol for different cycles

The AgNWs washed in ethanol were used to fabricate electrodes on PET substrates at room temperature. Figure 3 shows the relationship between the electrical performance and the optical transmittance of the electrodes. The deviations of measured sheet resistance values were usually less than ±1 % of the average value at each measuring point. When the AgNW loading decreased, the transmittance increased but so did the sheet resistance. However, washing drastically decreased the resistance while maintaining the transmittance. For example, the resistances from zero to four washing cycles were 195, 50.8, 32.0, 19.5, and 15.6 Ω sq^−1^, respectively, with the same transmittance of ca. 90 %. The results indicate that washing gradually removed the PVP layer leading to lower contact resistance between AgNWs. Importantly, the reductions in resistance were more notable at higher transmittance. For instance, at 85 % transmittance, the resistance dropped from 30 to 10 Ω sq^−1^ after four cycles of washing, while at 95 % transmittance, the drop is more dramatic, from 5 × 10^4^ to 93.0 Ω sq^−1^. At low transmittance, dense AgNW percolation networks leaded to a large number of conductive paths, and this balanced the deleterious effect of high contact resistance at the wire–wire junctions (see Fig. S2). At high transmittance, however, there were fewer conductive paths, so the effect of contact resistance was more pronounced. Indeed, according to the percolation theory of conductive networks [25], sheet resistance would dramatically increase at the critical concentration of AgNWs, i.e., percolation threshold, as the transmittance increased. In the present work, a thin PVP nanolayer made it possible to reduce percolation threshold and achieve low sheet resistance with a limited number of conductive paths. Moreover, within the measurement range, no clear correlation was observed between the thickness of the PVP nanolayer and the transmittance or haze of the fabricated electrode (see Fig. S3). This could be due to the thinness of the PVP nanolayer compared with AgNW and the high transmittance of PVP thin layer itself. Tailoring the thickness of the PVP nanolayer markedly improved the electrical characteristics of the electrodes with little or no effect on their optical properties. Summarily, it is necessary to wash AgNWs for four cycles using ethanol to reduce the PVP layer thickness to about 2.5 nm and achieve sheet resistance below 10 and 100 Ω sq^−1^ for transmittance below 82 and 95 %, respectively. However, since the reduction is lower after each cycle, the gains from washing more than four times were expected to be minor.

**Fig. 4:**
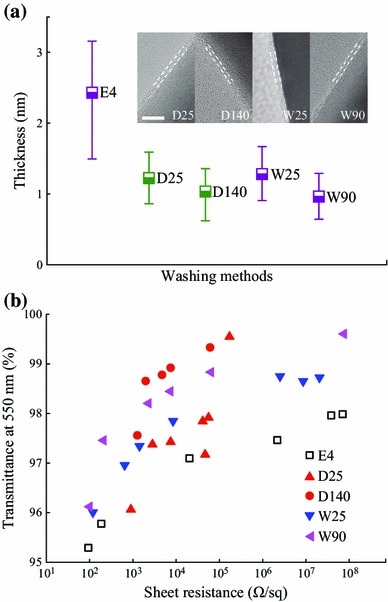
**a** Average thickness of tailored PVP nanolayer before and after further washing treatment in DMF or DI water.*Inset*: morphology of tailored PVP nanolayer after further dissolution treatment. *Scale bar* = 5 nm. **b** Sheet resistance of AgNW electrodes with high transmittance above 95 % at 550 nm wavelength, employing E4-AgNWs and AgNWs further treated in DMF or DI water

In order to further improve the contact between wires to achieve high conductivity, other solvents like DI water and dimethylformamide (DMF), which are better solvents for dissolving PVP (see Fig. S4), were used to wash the treated AgNWs. After washing with ethanol, E4-AgNWs were further treated in DMF or water, and the evolution of PVP nanolayer morphology was investigated as shown in Fig. 4. The average thickness decreased from 2.43 nm to approximately 1 nm. Washing at higher temperature (140 °C for DMF and 90 °C for DI water) led to greater reductions in thickness due to easier dissolution of PVP at higher temperature.

The sheet resistances of electrodes employing these four kinds of AgNWs were also compared with the ones employing E4-AgNWs (Fig. 4b). A similar pattern emerged that high-transmittance electrodes were more sensitive to changes in the thickness of the PVP nanolayer: for transmittance below 95 % at the wavelength of 550 nm, the sheet resistance was similar to that of ethanol-treated samples, while for electrodes with higher transmittance, a clear reduction in the sheet resistance was obtained. For example, the sheet resistance of electrodes using W90-AgNWs dramatically dropped from 2.1 × 10^6^ to 204 Ω sq^−1^ at the transmittance of 97.5 %. To the best of our knowledge, this result is the lowest value ever reported without any post-treatment and far exceeds that of ITO.

It should be noticed that the agglomeration of nanowires gradually emerged and finally precipitated when washing time in DI water or DMF was prolonged, corresponding to the excessive removal of PVP. The agglomeration degraded the dispersion of nanowires and thus the electrode performance at high transmittance. Therefore, it is important to maintain well-distributed nanowire percolation networks while reducing the contact resistance of individual wire–wire junction. In other words, the washing parameters should be carefully controlled to obtain PVP layer with a thickness optimal for peak electrical performance. The washing parameters included washing temperature, washing times, stirring speed, and the solvent type. Simple, time-saving or cost-effective washing pre-treatment could be easily achieved with different washing parameters to improve the electrical performance for various applications of AgNW transparent electrodes.

**Fig. 5:**
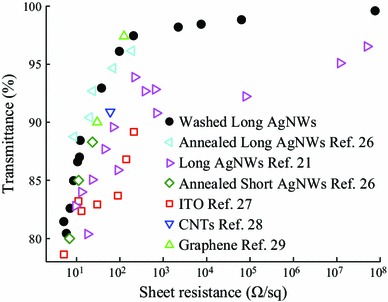
The optoelectrical performance of electrodes with W90-AgNWs compared with that of the other reported transparent electrodes

Figure 5 shows the comparison of the optoelectrical performances of the W90-AgNWs electrodes with those from previously published studies. It should be noted that our electrodes were fabricated at room temperature without any post-treatment. Electrodes produced by this simple washing method had much lower sheet resistance compared with long AgNWs [21] and showed similar or even lower values compared with AgNWs with an average length of 95.1 μm annealed at 250 °C for 2 h [26]. Clearly, the electrodes also performed better than electrodes with short annealed AgNWs [26] and better than other conductive materials such as ITO [27], CNTs [28], and graphene [29]. The high conductivity improved by pre-treatment enabled AgNWs to become excellent transparent electrode materials especially at high transmittance.

**Fig. 6:**
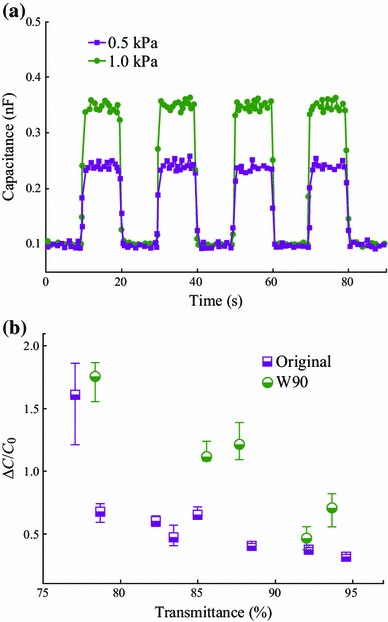
Electrodes with modified AgNWs were employed in capacitive pressure sensor application. **a** Capacitance response to pulsed pressure of 0.5 and 1.0 kPa, respectively. **b** Capacitance change Δ*C*/*C*_0_ of the pressure sensor with modified and unmodified AgNWs at various transmittances

The washed AgNWs not only improved the conductivity of the film electrodes, but also overcome the substrate limitation induced by the post-treatment and could be directly coated on various substrates with different shapes and properties. The W90-AgNWs were coated on the glass beaker, PET bottle, tissue paper, and bacterial cellulose substrate (Fig. S6). These AgNW films on different substrates all showed high conductivity without any post-treatment. The flat PET substrate-based electrodes with W90-AgNWs were incorporated into a capacitive pressure sensor for validation in a practical application. 5 wt % PVP solution in ethanol was coated on the AgNW electrodes and dried as the dielectric interlayer, and two pieces of sandwiched PET/AgNWs/PVP structures were stacked as shown in Fig. S7. This kind of structure could be fabricated in different shapes and sizes, on rigid or flexible substrates. First, the sensor employing two pieces of 93.7 %-transmittance AgNW electrodes was investigated (see Fig. S8). Pulsed pressures of 0.5 and 1.0 kPa were used to test the response of the capacitive sensor as shown in Fig. 6a. Under intermittent pressure stimuli of 0.5 kPa, the capacitance changed rapidly from ca. 0.1 to 0.24 nF, with good reproducibility throughout the test. When the pressure was increased to 1.0 kPa, a larger response of 0.35 nF was obtained with good stability. Under mechanical pressure, the distance between the overlapping areas of upper and lower electrodes decreased rapidly resulting in immediate changes in the capacitance [30]. Figure 6b compares the change in capacitance under a pressure of 0.3 kPa for sensors assembled using electrodes with different transmittances. The sensor using washed AgNWs showed a higher capacitance change Δ*C*/*C*_0_ (*C*_0_ is defined as the original capacitance without any pressure, and Δ*C* is defined as the difference between the capacitance with pressure and *C*_0_) at all transmittances compared to the sensor using untreated AgNWs, suggesting that the sensor using W90-AgNWs is more sensitive to external pressure due to the improved contact resistance between AgNWs. The high-performance sensor was successfully achieved with the pre-treated AgNWs, and this pre-treatment method also provides an effective and simple route to enhance the performance of other devices based on transparent electrodes.

## Conclusions

The conductivity of AgNW transparent electrode has been dramatically improved at high optical transmittance by a simple and rapid washing method without any post-treatment. In this washing pre-treatment process, increasing the number of washing cycles leads to the gradual reduction in the thickness of PVP layer and the corresponding decrease in the sheet resistance especially at high transmittance. Washing temperature and solvent type are also important factors in the pre-treatment process. Therefore, AgNW electrodes with sheet resistances of 15.6 and 204 Ω sq^−1^ at transmittances of 90 and 97.5 %, respectively, were produced without any post-treatment at room temperature. A capacitive pressure sensor based on the pre-treated AgNWs that performs with high sensitivity, reproducibility, and transparency, is demonstrated. The AgNW ink after washing pre-treatment also avoids the substrate limitation induced by the usual post-treatment and greatly expands the application of AgNW electrode on various substrates. 

## Electronic supplementary material

Below is the link to the electronic supplementary material.

Supplementary material 1 (DOC 4526 kb)
