# Supplementary material for: Silver Nanowire Electrodes: Conductivity Improvement Without Post-treatment and Application in Capacitive Pressure Sensors
Source: Nanomicro Lett. 2014 Nov 14;7:51–8. doi: 10.1007/s40820-014-0018-0 (PMC6223969; doi:10.1007/s40820-014-0018-0)
Supplement: Supplementary file 1 — Supplementary material 1 (DOC 4526 kb) [file 40820_2014_18_MOESM1_ESM.doc]

Supporting Information for

**Silver Nanowire Electrodes: Conductivity Improvement without Post-treatment and Application in Capacitive Pressure Sensors**

Jun Wang^1,2^, Jinting Jiu^2^,*, Teppei Araki^2^, Masaya Nogi^2^, Tohru Sugahara^2^, Shijo Nagao^2^, Hirotaka Koga^2^, Peng He^1^,*, Katsuaki Suganuma^2^

^1^State Key Laboratory of Advanced Welding and Joining, Harbin Institute of Technology, Harbin 150001, People’s Republic of China

^2^The Institute of Scientific and Industrial Research, Osaka University, Ibaraki, Osaka 567-0047, Japan

*Corresponding author. E-mail: [jiu@eco.sanken.osaka-u.ac.jp](mailto:jiu@eco.sanken.osaka-u.ac.jp) (Jinting Jiu), [hithepeng@hit.edu.cn](mailto:hithepeng@hit.edu.cn) (Peng He)


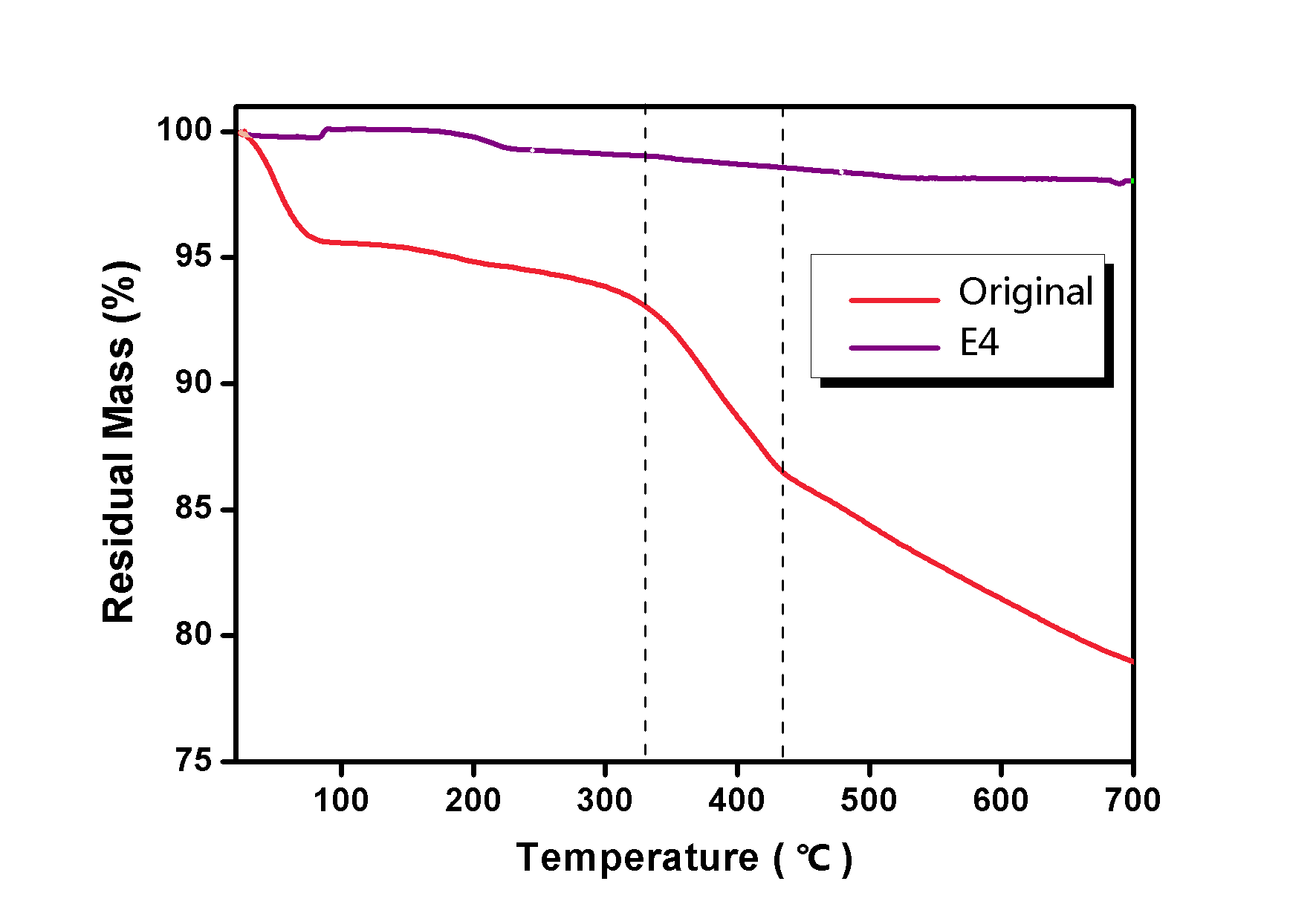


**Fig. S1** Thermalgravimetric analysis of original AgNWs and after 4 cycles washing treatment in ethanol, respectively. For the original AgNWs sample, the weight loss was much higher than the E4-AgNWs during the same thermal process, which indicated more residual organics before washing treatment. In particular, during 300-400 °C, the rapid weight-loss was usually mainly attributed to the evaporation and decomposition of residual PVP. The mass loss after 400 °C corresponds to the gradual burning and decomposition of the organic by-products in the polyol process


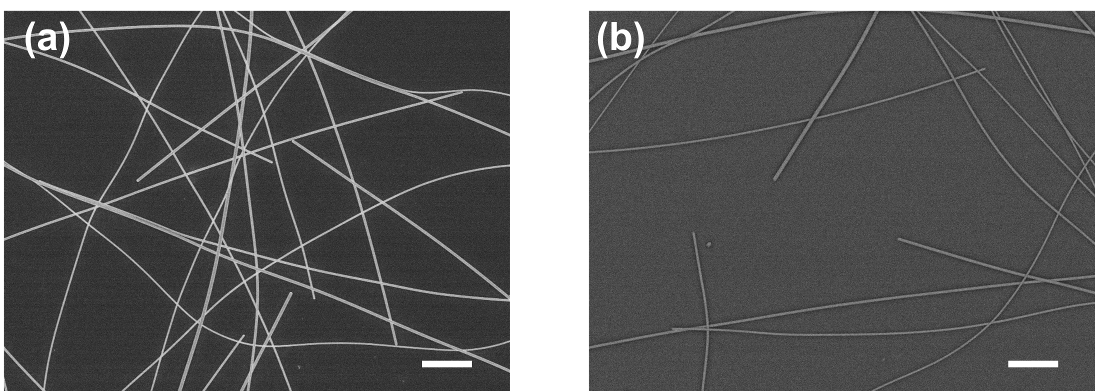


**Fig. S2** The typical AgNW networks with lower and higher transmittance, respectively. **a** Dense AgNW percolation networks with a large number of conductive paths, at transmittance of ca. 85 %, 550 nm wavelength. **b** Less AgNWs and conductive paths with transmittance of ca. 90 %. Scale bar = 2 μm


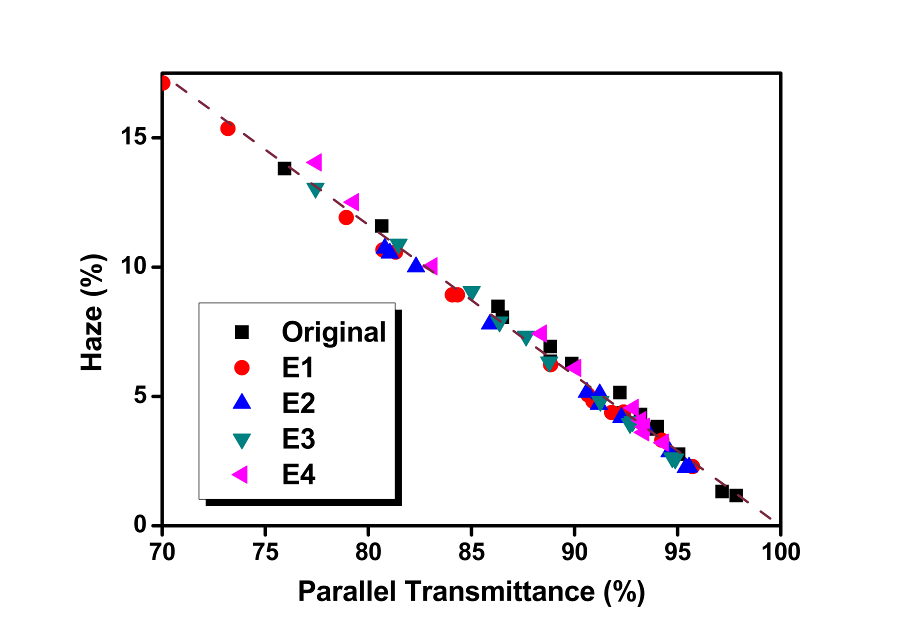


**Fig. S3** Haze vs. parallel transmittance of electrodes employing AgNWs before and after washing in ethanol for 1, 2, 3, 4 cycles, respectively. It could be observed that the five groups of data points almost distributed along the same straight line, i.e. followed the same linear relationship of the two optical parameters. It indicated that the haze value of the certain AgNW electrode would keep stable at the same transmittance even the thickness of adsorbed PVP layer dramatically changed comparing to the original one


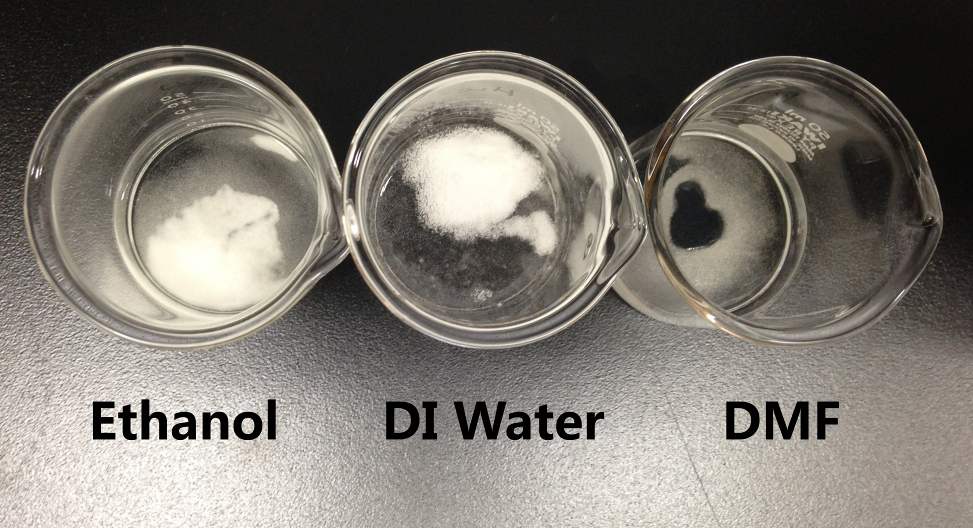


**Fig. S4** Same amount of PVP powders (0.8 g) were respectively dissolved in three solvents, i.e. ethanol, DI water and DMF, with stirring of 300 rpm at room temperature. All the 0.8 g PVP powders were added into the solvents at the same time. It took 90, 60, and 40 min for complete disolusion in ethanol, DI water and DMF, respectively. It indicated the PVP used in this study has higher solubility in DI water and DMF, and the two solvents could further remove PVP nanolayer. However, it also increased the risk of nanowire agglomeration due to PVP nanolayer would be dissolved more rapidly in DMF and DI water


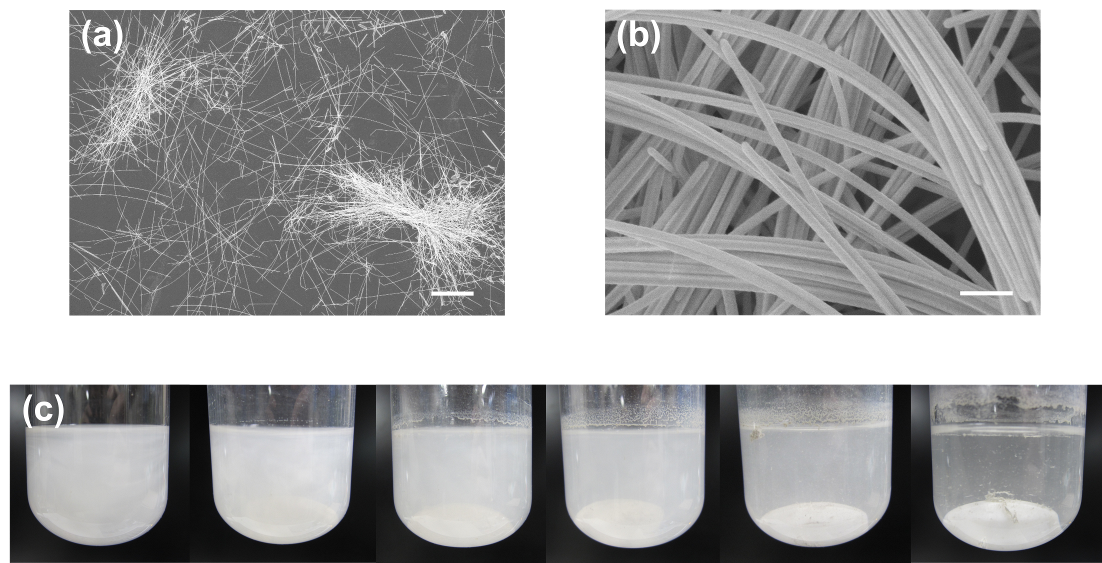


**Fig. S5** **a** Emerged agglomerations in AgNW suspension. Scale bar=20 μm. **b** Morphology of the agglomerated nanowires. Scale bar = 500 nm. **c** AgNW suspension treated by washing method in DI water at 90 °C with stirring for 0, 15, 20, 35, 50, and 65 min, respectively. AgNW agglomerations emerged after PVP nanolayer was excessively removed. The nanowires tended to adsorb with each other like cables and then twist together **b**. If the washing time was extended, almost all of the nanowires would gradually join in the agglomerations, and the suspension was gradually clarified in the meantime. The agglomerations would undermine the electrode performance as many nanowires gathered together, instead of dispersing uniformly on the substrate. Therefore, the sheet resistance will increase due to the lack of conductive paths


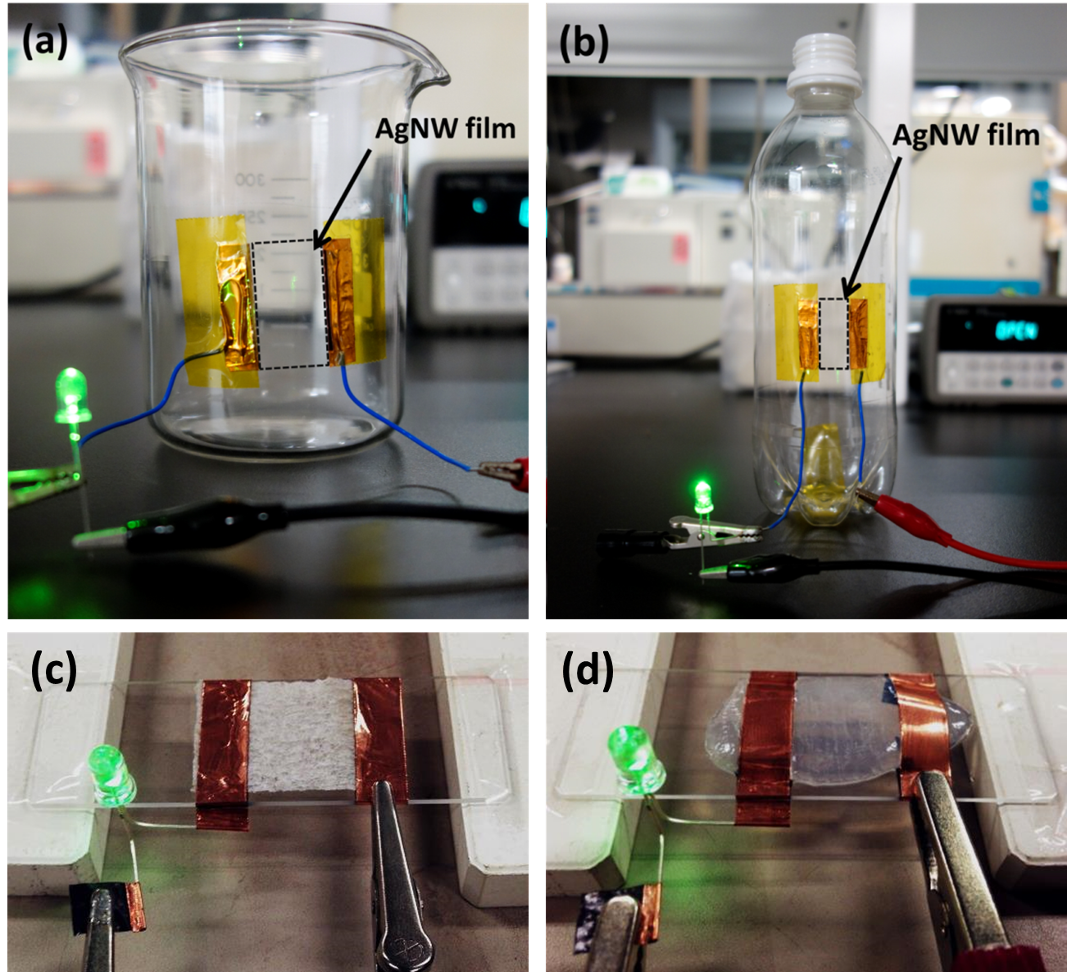


**Fig. S6** As-washed AgNW films coated on a glass beaker **a** and on a PET bottle **b** showed high transmittance and conductivity in circuit connected with a light-emitting diode


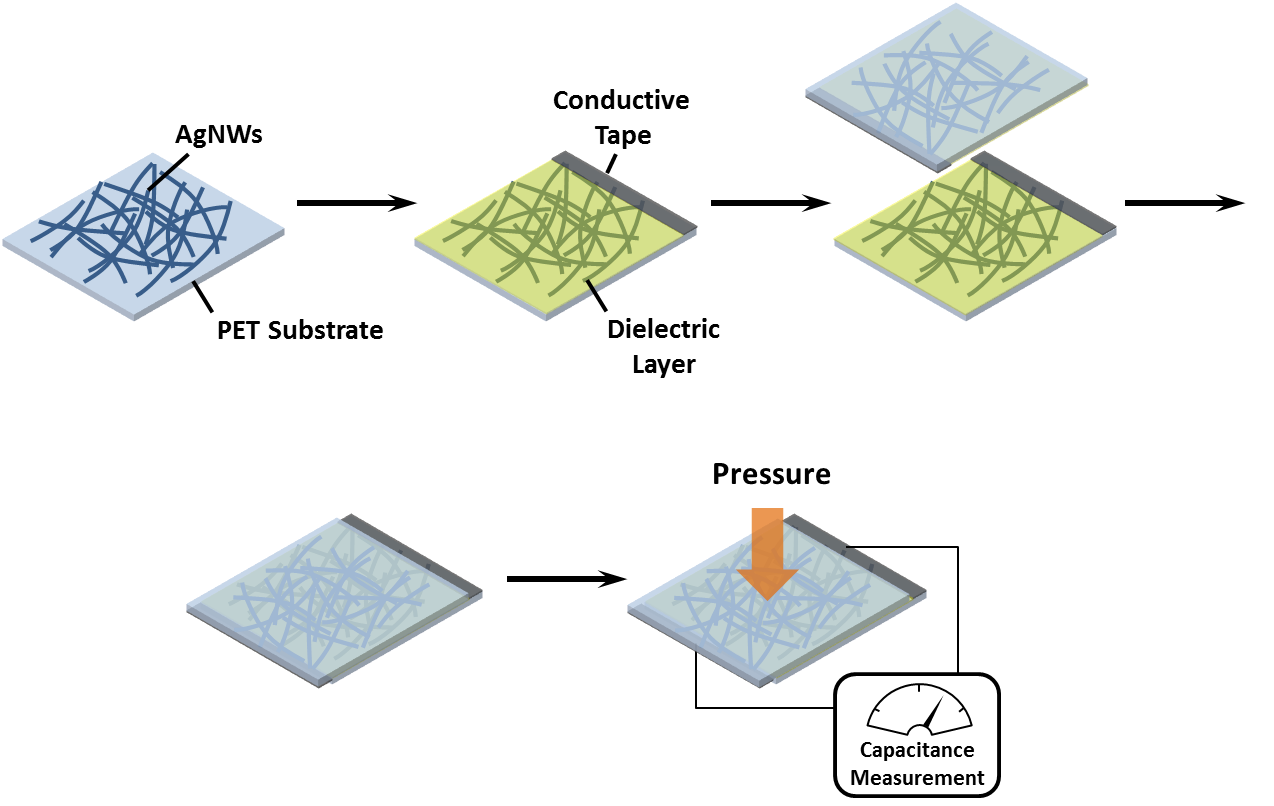


**Fig. S7** Schematic of capacitive pressure sensor based on silver nanowire (AgNW) transparent electrodes. Two pieces of AgNW electrodes employing polyethylene terephthalate (PET) substrates were spin-coated with PVP solution in ethanol and dried in air. The formed PVP films were regarded as dielectric interlayer as the two pieces of sandwiched structures were assembled with each other. Conductive tape was also applied in the structure for signal collection and measurement.


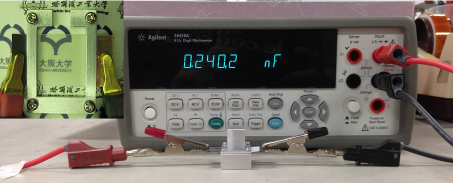


**Fig.** **S8** The experimental setup of capacitance response measurement. Inset: Transparency of the capacitive pressure sensor involving both AgNW electrodes with transmittance of 93.7 % at 550 nm wavelength.


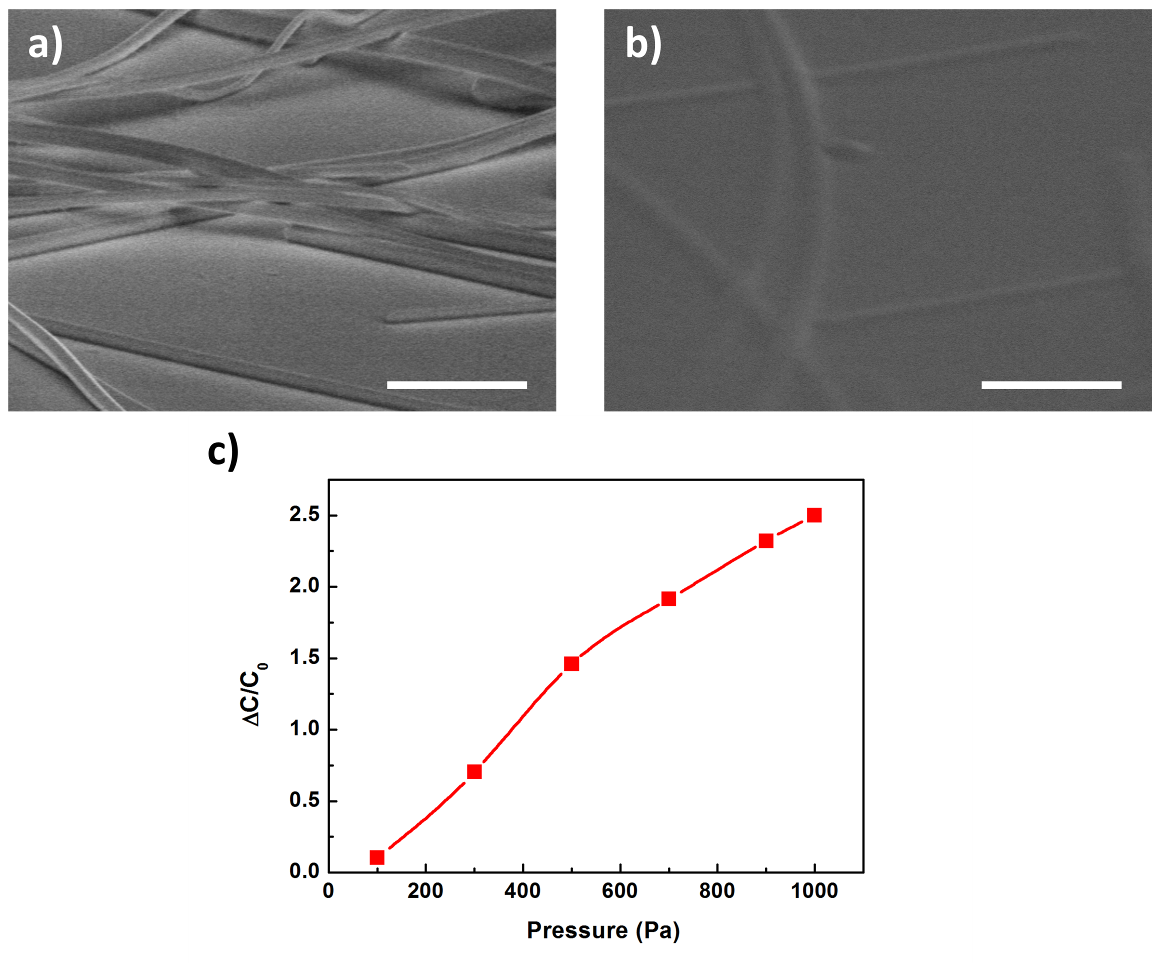


**Fig. S9** The microstructure of **a** the tilted AgNW electrode and **b** the PVP layer coated on AgNW electrode. a) shows the rough surface of the AgNW electrode and **b** shows the patterned surface of PVP layer due to the roughness of AgNW film. **c** The capacitance change of the sensor with differnent applied pressure
